# Cognitive and affective perspective-taking in conduct-disordered children high and low on callous-unemotional traits

**DOI:** 10.1186/1753-2000-2-16

**Published:** 2008-07-07

**Authors:** Xenia Anastassiou-Hadjicharalambous, David Warden

**Affiliations:** 1 Department of Psychology, University of Strathclyde, 40 George Street, Glasgow, G1 1QE, UK; 2 Department of Psychology, University of Nicosia, 46 Makedonitissas Avenue, P.O. Box 24005, 1700, Nicosia, Cyprus

## Abstract

**Background:**

Deficits in cognitive and/or affective perspective-taking have been implicated in Conduct-Disorder (CD), but empirical investigations produced equivocal results. Two factors may be implicated: (a) distinct deficits underlying the antisocial conduct of CD subgroups, (b) plausible disjunction between cognitive and affective perspective-taking with subgroups presenting either cognitive or affective-specific deficits.

**Method:**

This study employed a second-order false-belief paradigm in which the cognitive perspective-taking questions tapped the character's thoughts and the affective perspective-taking questions tapped the emotions generated by these thoughts. Affective and cognitive perspective-taking was compared across three groups of children: (a) CD elevated on Callous-Unemotional traits (*CD-high-CU*, *n *= 30), (b) CD low on CU traits (*CD-low-CU*, *n *= 42), and (c) a 'typically-developing' comparison group (*n *= 50), matched in age (7.5 – 10.8), gender and socioeconomic background.

**Results:**

The results revealed deficits in *CD-low-CU *children for both affective and cognitive perspective-taking. In contrast *CD-high-CU *children showed relative competency in cognitive, but deficits in affective-perspective taking, a finding that suggests an affective-specific defect and a plausible dissociation of affective and cognitive perspective-taking in *CD-high-CU *children.

**Conclusion:**

Present findings indicate that deficits in cognitive perspective-taking that have long been implicated in CD appear to be characteristic of a subset of CD children. In contrast affective perspective-taking deficits characterise both CD subgroups, but these defects seem to be following diverse developmental paths that warrant further investigation.

## Background

Most theories hold that, although inhibition of antisocial conduct is primarily mediated by affective empathy (i.e. vicarious affective responsiveness), cognitive dimensions of empathy such as perspective-taking skills also play a substantial role. For instance, it has been suggested that the ability to differentiate among and identify others' affective states, and the ability to take their cognitive and affective perspective are prerequisites for empathising [1,2] and thereby inhibiting antisocial conduct. Hoffman, in his influential developmental model of empathy [3], gives primacy to the affective dimensions of empathy, postulating that the observation of distress in others triggers an innate 'empathic distress' in the child, even before s/he has the cognitive capacity to differentiate 'other' from 'self'. However, he also proposes that intentional moral conduct is determined by the capacity to take another's perspective. This view dovetails nicely with Piaget's [4] theoretical work which stresses the importance of perspective-taking capacity for enabling an individual's anticipation of others' behaviour and reactions, therefore leading to smoother interpersonal relationships. Blair and colleagues [5], suggest that persistent antisocial conduct results from an early dysfunction within the 'Violence Inhibition Mechanism', which is involved in the control of aggression in the normally developing child.

If perspective-taking is important for engaging in intentional moral conduct [3], or for facilitating social functioning [4], it is likely that deficits in the ability to understand another's cognitive and affective perspectives may be implicated in persistent antisocial conduct. For instance, Gough [6] and Hare [7] have long ago suggested that a history of antisocial behaviour results from a deficiency in perspective-taking. Empirical studies, however, examining cognitive and/or affective perspective-taking in children with conduct problems, have produced equivocal results depending on both the population tested and the perspective-taking measures employed.

Across the early studies, one of the most widely used assessments of perspective-taking has been the Flavell and colleagues [8] role-taking task. This measure consists of cartoon story sequences which the participant must describe, firstly from the central character's viewpoint, and then as the bystander in the story might see it. The bystander does not witness prior events which the central character has experienced, but only witnesses the resultant behaviour. In this measure, high scores are given to participants who successfully withhold this privileged information when asked for their description of the bystander's perspective. Using this measure (or slight modifications thereof), delinquent child and adolescent samples were reported to have marked deficits in the ability to successfully adopt the cognitive perspectives of others [9-11]. Whether these findings with delinquent samples apply to conduct-disordered (CD) populations remains unclear. Although most delinquents would meet the psychiatric criteria for CD, delinquency is a legal term used to portray children and adolescents identified by the legal system as having broken the law.

Empirical data on the perspective-taking abilities of CD children are scarce. In a study with institutionalised CD children, and utilising the Flavell et al. role-taking task, Chandler, Greenspan and Barenboim [12] reported inferior cognitive perspective-taking skills in CD children compared to controls. Institutionalised CD boys (aged 10) were reported to be inferior to typically-developing boys in cognitive perspective-taking in a study by Waterman and colleagues [13]. However, this study utilised the Flavell et al. perspective-taking logic task in which children are required to provide rationales for a guessing game strategy. Rationales are scored in terms of the extent to which the child recognises another's ability to take the child's own strategy into account. However, this task, apart from being cumbersome, mostly taps problem-solving skills rather than cognitive perspective-taking.

Over the last two decades, a broadly used paradigm for the assessment of cognitive and affective perspective-taking has been the 'false-belief ' task. False-belief tasks, often referred to as 'theory of mind' tasks, were initially intended to tap the ability to attribute mental states in children up to the age of five (first-order false-beliefs tasks) [14-17]. Subsequently, further tasks have been developed, with increased cognitive requirements (usually designated as 'second-order' and 'advanced' tasks), intending to tap perspective-taking in children throughout childhood and adolescence [18-22]. The common feature of these perspective-taking tasks is the formation of a false-belief about a social situation. One character is privy to information of which the second character is not aware. The task assesses the extent to which a child is aware of the differing thoughts and resulting emotions that the story characters have of the same situation, based on their differing perspectives. Studies on the psychometric properties of the theory-of-mind tasks report that these tasks report good test-retest reliability and internal consistency [23].

Employing a false-belief paradigm, Happé and Frith [24] reported no evidence of deficits in inferring others' thoughts in CD children (6–12 years) recruited from a day school for children with Emotional and Behavioral difficulties (EBD), in comparison with 'typically-developing' controls (7–9 years). Happé and Frith, however, utilised a small sample size (18 CD children and 8 controls) and a first-order task which, if used with individuals whose mental age is more than six years, is subject to ceiling effects [21]. Therefore, it seems plausible that the lack of perspective-taking deficits in the CD sample in the Happé and Frith study is due to the relative simplicity of the measures.

In a correlational study with a normative sample (11–13 years), Sutton and colleagues [25] used an advanced theory-of-mind paradigm and found no evidence of link between the ability to infer others' thoughts/emotions and conduct problems (as measured by a self-report comprising all but one of the diagnostic criteria for CD [26]. As a guide to the level of conduct problems in the sample, it was reported that 10% satisfied CD criteria. However, as self-report assessments were used, and persistence of conduct problems was not accounted for, these findings might not generalise to CD populations.

To summarise thus far, the evidence reviewed has either supported the hypothesised negative association between affective and/or cognitive perspective-taking and antisocial conduct, or corroborated the null hypothesis. However, there is further line of empirical evidence, with both normative and CD populations, that contradicts theoretical speculations. For instance, in a study utilising a false-belief paradigm with a normative sample, Sutton and colleagues [27] found that, on combined cognitive and affective perspective-taking scores, 'ringleader' antisocial children outperformed not only their 'followers' (those who helped them) and their victims, but also the prosocial children. When affective and cognitive perspective-taking were considered independently, the 'ringleader' antisocial children outperformed the followers in affective perspective-taking but no group differences were observed in cognitive perspective-taking. These findings may not necessarily apply in CD populations. Nevertheless, they seem to suggest a possibly distinct operation of cognitive and affective perspective-taking across diverse subgroups of children with conduct problems. In a further normative study challenging conceptual expectations, and suggestive of a differentiated operation of affective and cognitive perspective-taking, Silvern and colleagues [28] reported that, among 10–11 year-old boys, cognitive perspective-taking superiority was associated with relatively more severe antisocial behaviour. In contrast, Waterman et al., [13] utilising a normative sample and a sample of institutionalised CD boys, reported no significant correlation between antisocial behaviour and cognitive and affective perspective-taking across the normative sample, whereas, in the CD sample, affective, but not cognitive, perspective-taking superiorities were associated with higher antisocial behaviour. Finally, Happé and Frith [24] reported that CD children demonstrated advanced mentalising abilities in domains of antisocial behaviour (lying, cheating, teasing, bullying) that presuppose well functioning cognitive perspective-taking abilities.

These inconsistent findings across investigations seem to be the outcome of a substantial heterogeneity within children exhibiting conduct problems, possibly coupled with a distinct operation of cognitive and affective perspective-taking abilities. Consequently, the present study aims to investigate a possible heterogeneity of CD children and a distinct operation of cognitive and affective perspective-taking across CD subgroups. A growing body of empirical literature suggests that CD children form a diverse group whose subgroups differ with respect to comorbid symptomatology, developmental trajectories, types of behaviors exhibited, and the causes of behavior problems [29,30].

With respect to comorbid symptomatology, subsets of CD have comorbid symptoms of Attention Deficit Hyperactivity Disorder (ADHD, 65 to 90 percent) [31], depression (15 to 31 percent) [32], anxiety (22 to 33 percent for community samples and 60 to 75 percent in clinic samples) [32], and Post Traumatic Stress Disorder (PTSD) symptoms resulting from a high prevalence of trauma histories in their life [33].

Frick and colleagues [34] classified CD subgroups in terms of the presence of callous-unemotional (CU) traits (e.g. lack of guilt, lack of empathy), an approach which is analogous to adult conceptualizations of psychopathy. The logic behind this classification system derives from studies revealing distinct correlates for the subsets of CD children who also show high levels of CU traits (*CD-high-CU*) compared to those who do not (*CD-low-CU*).

*CD-high-CU *children, who are primarily characterized by proactive forms of aggression [35], have shown substantial evidence of deficits in emotion processing such as decreased orienting to affective stimuli [36,37] low fearful inhibition [38,39] and reduced vicarious affective responsiveness [40] underlined by underactivity in the sympathetic autonomic nervous system [41]. All these findings may be suggestive of affective-specific deficits in *CD-high-CU *children. In *CD-low-CU *children, on the other hand, reactive rather than proactive patterns of aggression have been reported [42,43] and their lack of impulse control has been related to a diverse set of interacting causal factors [34] such as social information processing deficits [44], dysfunctional family background [45,46] and verbal intelligence deficits [47]. Perspective-taking deficits in this group may therefore be cognitive specific.

Consequently, the present study set out to compare affective and cognitive perspective-taking in three groups of children a) *CD-high-CU*, b) *CD-low-CU*, and c) an age, gender and socioeconomic background (SES) matched 'typically-developing' comparison group. A second order false-belief paradigm, utilising cartoon strip stories, was designed to assess both cognitive and affective perspective-taking. A series of questions was devised to respectively elicit participants' awareness of the thoughts of the cartoon characters and the emotions generated by these thoughts. This methodology would test whether any specific group manifested a dissociation between inferring others' thoughts and their consequent emotions. Based on the line of reasoning described above, it was predicted that *CD-low-CU *children will present deficits in both cognitive and affective perspective-taking (given that understanding others' emotions depends first on understanding their thoughts), relative to the 'typically-developing' comparison group. *CD-high-CU *children will present deficits in affective, but not in cognitive perspective-taking, relative to the 'typically-developing' comparison group. Further, this study utilized a verbal skills measure to account for plausible confounding effect of verbal ability.

## Methods

### Participants

The CD sample was recruited in two phases. In phase one, following written parental consent, an initial sample of children meeting CD diagnostic criteria [26] was identified on the basis of diagnostic information contained in their files in six different settings. The six settings are as follows: Four schools offering day special education programs for children with Emotional and Behavioral Difficulties (EBD, 35%), one school offering residential intervention to children with severe EBD (41%) and a university based diagnostic service that provides psychological evaluations for children with EBD (24%). This first phase yielded an initial sample of 163 CD children that were predominantly boys (96%), English-speaking (100%), of white ethic origin (100%). From this initial CD sample, children diagnosed with severe learning disabilities (*n *= 5) or with a pervasive developmental disorder (*n *= 4) were excluded from follow up assessments.

In the second phase of recruitment, the sample of 154 was further screened to determine the degree of their conduct problems (evaluated on the *Conduct Difficulties Rutter Teacher Scales for School-age Children *[48]), and to identify a group of CD children elevated on Callous-Unemotional traits (evaluated on the CU subscale of the *Antisocial Process Screening Device (APSD) *[49], and a group that would score low on this measure. CD children whose score on the CU subscale fell in the upper quartile of the screened sample were placed in the CD group high on CU traits (*CD-high-CU*). CD children whose score fell on, or below, the 50^th ^percentile of the screened sample were placed in the CD group low on CU traits (*CD-low-CU*).

For the clinic-referred children, evaluations were completed by the individual child's form teacher and the primary caregiver (usually the mother). For the institutionalized children, evaluations were completed by the individual child's form teacher and the primary caregiver or a staff professional specialized in social work. These professionals had daily contact with the children, regular contact with their parents, and access to extensive information contained in their files. Information from these two informants was combined using the approach recommended by Piacentini and colleagues [50] in which a symptom is considered to be present if reported by any single informant. This approach takes into consideration that each informant might has a different but still valid perspective on the symptom in question and therefore the unique information provided by each informant is preserved. Further, given that CU traits are not socially desirable, there is an increased possibility that there would be a tendency of some informants to underreport such traits, and at the same time a decreased possibility to overreport these traits. Consequently, considering CU traits to be present only when both informants would report them would not be justifiable.

The sample of controls was recruited from state schools in areas surrounding the settings from which the CD groups were selected. On the basis of their evaluation on the conduct difficulties scale [48] completed by their parents and their teachers, four control children met CD criteria and were excluded. With a view to forming balanced groups in terms of age, gender and SES we excluded from the sample the 10 control children with the highest SES. The demographic and diagnostic characteristics of the sample are provided in Table 1.

**Table 1:** Demographic and diagnostic characteristics of the sample

**Characteristic**	**CD-high-CU** ** (n = 30)**	**CD-low-CU** ** (n = 42)**	**Control** ** (n = 50)**	**Statistic**
Age. *M *(*SD*)	9.37 (1.2)	9.00 (.98)	9.04 (.70)	*F *(2, 119) = 1.6
SES. *M *(*SD*)	33.23 (18.20)	36.10 (19.53)	38.32 (20.59)	*χ*^2^(2, 122) = 2.78
Gender. % female	3.3%	4.8%	6%	*χ*^2 ^(2, 122) = .48
CU Traits. *Mdn *(*IQR*)	10 (1)_a_	4 (4)_b_	3.5 (3)_b_	*χ*^2 ^(2, 122) = 69.62**
Conduct problems. *Mdn *(*IQR*)	18 (3)_a_	15 (5.25)_b_	4 (5)_c_	*χ*^2^(2, 122) = 91.53**
CD/ODD diagnosis (%)	100	100	0	
ADHD diagnosis (%)	33.3%	28.57%	14%	*χ*^2^(2, 122) = 4.68
Anxiety diagnosis (%)	16.67%	26.19%	14%	*χ*^2^(2, 122) = 2.34
Depression diagnosis (%)	20%	16.67%	4%	*χ*^2^(2, 122) = 5.59
Expressive language. *Mdn*(*IQR*)	93 (20.25)	90.5 (27.25)	95.5 (12)	*χ*^2^(2, 122) = 2.42

*Note*: Level of conduct problems was determined by the Revised Rutter Teacher conduct difficulties subscale (Hogg et al., 1997); ODD = Oppositional Defiant Disorder; ADHD = Attention Deficit and Hyperactive Disorder; ADHD, CD/ODD, Anxiety and Depression diagnoses were determined by diagnostic information contained in the participants files. Expressive language was determined by the *Word Definition Test *(WD-BASII) British Ability Scales II; SES (socioeconomic status) was determined by the Duncan's socioeconomic index (Hauser & Featherman, 1977); *M*: Mean, *Mdn*: Median; *IQR *= Interquartile Range; *SD *= Standard Deviation; Effects on CD diagnosis could not be calculated because no diagnoses were present in the control group; Medians in the same row that do not share subscripts differ at *p *< .05 in the Mann-Whitney *U *procedure.

**p < .001

### Measures

#### Conduct Difficulties Subscale of the Revised Rutter Teacher Scales for School-age Children [48]

This is a 10-item subscale of the Rutter scales that were developed in the UK to detect conduct problems among children aged 3–16, and have been widely used and evaluated [51]. The correlation of the scores assigned by the two informants suggested reasonable consistency (*r *= .68, *p *< .001). The Rutter scales are fairly brief to complete yet correlate well [51] with the Child Behavior Checklist [52].

#### Antisocial Process Screening Device [49]

The APSD, formerly known as the Psychopathy Screening Device [53], is a 20-item behavior rating scale developed to measure CU traits, narcissism and poor impulse control in children. Three different subscales deriving from factor analysis [53] have been developed: a 6-item 'Callous-Unemotional' (CU) factor tapping unemotional interpersonal style (e.g. is unconcerned about the feelings of others); a 6-item 'Narcissism' factor tapping narcissistic traits (e.g. thinks s/he is more important than others), and; a 5-item 'Impulsivity' factor tapping impulsive behaviors (e.g. 'acts without thinking'). The CU dimension has proven to be the most stable dimension of the APSD across multiple samples [53]. It had an internal consistency of .76 in the full screening sample. In the current sample, the correlation of the ratings of the two informants for the CU subscale was .59, suggesting reasonable consistency.

#### Word Definitions Test of the British Ability Scales II [54]

This measure was included as a control measure to examine whether any differences in perspective-taking could partly be explained by differences in verbal ability. During administration, tentative scores were assigned in order to use the decision point and alternative stopping point rules. After testing, the detailed scoring procedure of the Administration and Scoring Manual of the British Ability Scales [54] was followed. Age-corrected T-scores were used in the analysis of the data.

#### Affective and Cognitive Perspective-Taking

Two second-order false-belief stories [23] were modelled on previous studies of perspective taking [18,19]. The stories were developed around social situations with which the children would be familiar, but had a degree of situational complexity. The common feature in these 'social stories', that allowed perspective-taking ability to be assessed, was the differing perspectives and false beliefs that the main characters had about the situation and each other's cognitions. Each story was accompanied by a three-picture storyboard (strip cartoon), which elucidated the critical features of the story. For each story, a set of questions was constructed, comprising (a) comprehension questions – to assess children's understanding of the factual content of the story, (b) cognitive questions – to assess children's interpretations of the different cognitive perspectives and false beliefs of the story characters, and (c) affective questions – to assess children's ability to both describe and explain the emotional responses of the story characters which were based on the characters' false beliefs. One example of the stories is shown below, with its accompanying set of questions; the second story is available from the first author on request. Studies of the psychometric properties of second-order false-belief tasks show good test-retest reliability and internal consistency, with very strong test-retest correlations between aggregate scores, for children of all levels of ability [23].

Birthday Present: ***Louise ****has asked her sister Mary to give her a CD of her favourite group Boyzone, for her birthday. The day before her birthday, Louise accidentally knocks Mary's bag on the kitchen floor. Some red wrapping paper and a CD fall out. The CD is All Saints, a group Louise hates. Louise puts them back in Mary's bag and goes to her room. Then Mary comes into the kitchen with a new CD of Boyzone, and wraps it in the red wrapping paper. Next day, Mary gives Louise her birthday present, wrapped in red paper. Before she opens it Louise says, 'I really like All Saints now'. Then she unwraps the paper, and finds a CD of Boyzone inside*.

Comprehension questions:

e.g. What did Louise want for her birthday?

Cognitive perspective-taking:

e.g. *Why did Louise say to Mary 'I really like All Saints now'?*

Affective perspective-taking:

e.g. How did Mary feel when Louise said she likes All Saints? – Why?

#### Establishing scoring criteria for the false-belief task

A series of steps was followed to establish scoring criteria for the false-belief paradigm. In the initial stage, a sample (n = 30, 10 for each group) of children's responses was discussed by a panel of independent judges (three researchers in the field of developmental psychology), who were unaware of both the hypothesis being tested and the group origin of the data. Using as a template the coding scheme developed by Warden and colleagues [22] which had demonstrated an interjudge agreement of 90–100%, the judges formulated the following coding scheme. Cognitive perspective-taking responses were assigned a score ranging from 0 to 2. A score of 2 was assigned if a child's response demonstrated an understanding of the false-belief and/or highlighted the differing perspectives of the characters in the story. If an answer was based on a purely descriptive understanding of the social story, giving no justification in terms of another person's perspective, a score of 1 was assigned. This score was also given to factually correct answers which were poor in reasoning and/or lacked any detail or elaboration. A score of 0 was assigned to incorrect and irrelevant answers and when a child was unable to give an answer. Across the two stories used in the false-belief task, there was a total of six questions assessing cognitive perspective-taking yielding a maximum potential score of 12.

Affective perspective-taking responses were assigned a score ranging from 0 to 2. Irrelevant and non-answers were assigned a score of 0. Responses that used moderately relevant emotional descriptors and were justified with reference to the protagonist's immediate situation rather than to the false-belief got a score of 1. A score of 2 was assigned to responses that involved a highly relevant emotional descriptor and demonstrated an awareness of either: a) the false-belief, or; b) the confounded expectation of the protagonist. There was a total of six questions assessing affective perspective-taking, yielding a maximum potential score of 12.

In order to validate these coding criteria, a second panel of independent judges (research psychologists, *n *= 12), who were naïve to the hypothesis being tested and the group origin of the data, scored a sample of children's responses (*n *= 60, 20 for each participant group). Interjudge agreement, calculated for each group separately to ensure that agreement was not significantly lower for any particular group, was 85% or better for each group. The coding scheme described above was then used (by the first author) to score the responses of all the participants. Scoring was blind to the group origin of the data.

Given the element of subjectivity inherent in the judgment of the responses, further validation of the scoring was deemed to be necessary. Therefore, a random sample of 20% of coding sheets from each group was assigned to a second judge who was naïve to the hypothesis being tested and the group origin of the data. The degree of interjudge agreement was calculated (using the weighted Kappa procedure) for each group separately: agreement for affective perspective-taking was 87.5% (Cohen's Kappa = .78) and for cognitive perspective-taking 90% (Cohen's Kappa = .8) or better for each group.

### Procedure

#### Identification of CD children, familiarisation and sample identification

Given the nature of CD children's difficulties, it was important to familiarize them with the investigator (XAH). Over a period of two months, and before conducting any assessments, the investigator spent two days a week in each of the five participating EBD institutions. Such familiarization was not considered necessary for the comparison group. Upon obtaining parental consent, and during the period of familiarization with the CD children, the informants completed the Conduct Difficulties Subscale and the CU subscale in their own free time. Each scale took approximately five minutes to complete.

#### Assessment of cognitive and affective perspective-taking

All participating children were interviewed individually, in a quiet room, adjacent to their classroom. The false-belief stories were introduced as follows: '*I am going to read out some stories and questions and I'd like you to listen carefully and help me with the questions at the end of each story*.' Whilst reading each story, the experimenter identified the relevant protagonist by pointing. The cartoon strip remained in front of the child throughout the presentation of the questions to minimise memory requirements. The two control questions were presented first. On the occasions that the child failed to answer one of the control questions, the story was read out again. No child failed the control questions after the second reading. The questions assessing the affective and cognitive perspective-taking were then presented in the chronological order in which the events referred to had occurred. The order of presentation of the two stories was counterbalanced across participants, but the order of questions was constant.

For affective perspective-taking questions, the children were reminded that they had to say how they believed the story protagonist felt and not how they would feel in the protagonist's place. Children's verbal responses were recorded in full on scoring sheets for subsequent analysis. On the occasions when a child could not answer any question, the question was re-read and the child was prompted to make sure that s/he was unable to answer. Any '*don't know*' responses were noted on the response sheet. Positive comments were made throughout the testing sessions to encourage the child, but no feedback was given about the correctness of his/her responses. Administration was adjusted to suit the requirements of each participant, with repetitions and interruptions when necessary; the duration of the sessions therefore varied from approximately 8 to 20 minutes.

#### Assessment of verbal ability

The *Word Definitions Test *was administered to the children on an individual basis in a quiet room of their school. Responses were noted verbatim but also tape-recorded for subsequent analysis. This assessment took around 15 minutes depending on the child's verbal ability.

The order of the tasks was counterbalanced across participants.

## Results

### Statistical analysis

All data are expressed as the mean (*SD*) following the Shapiro-Wilk test for the normality of distribution. For data that violated the assumptions for parametric analysis (i.e. equality of variance and normality of distribution) non-parametric analysis was carried out and these data are expressed as the median (Interquartile range, *IQR*). For parametric data differences were determined by ANOVAs followed by Tukey's *HSD *procedures for the pairwise comparisons. For non-parametric data Kruskal-Wallis tests followed by Mann-Whitney *U *tests for the pairwise comparisons. Frequency data were analysed using the chi-square (*χ*^2^) statistic. Statistical significance was declared at *p *< .05.

### Demographic and Diagnostic Characteristics

To evaluate the equivalence of the three groups, a comparison was made of the demographic and diagnostic characteristics. As presented in Table 1, the three groups did not differ with respect to age, gender, SES, ADHD, depression and anxiety diagnosis and expressive language. Group differences were observed on the level of conduct problems and CU traits. On the level of conduct problems, the *CD-high-CU *children exceeded both the controls (*z *= 7.49, *p *< .001) and the *CD-low-CU *children (*z *= 4.01, *p *< .001); *CD-low-CU *children exceeded the controls (*z *= 8.04, *p *< .001). On CU traits, the *CD-high-CU *group exceeded both the controls (*z *= 7.53, *p *< .001) and the *CD-low-CU *(*z *= 7.29, *p *< .001) children.

### Affective and Cognitive Perspective-taking

As described in Table 2 there was a statistically significant difference between the affective perspective-taking of the three groups. Pairwise comparisons showed that the *CD-low-CU *group was outperformed by both the control (*z *= -5.40, *p *< .001) and *CD-high-CU *(*z *= -2.19, *p *< .03) groups. *CD-high-CU *group was outperformed by controls (*z *= -2.27, *p *< .02).

**Table 2:** Group comparisons on affective and cognitive perspective-taking.

**Characteristic**	**CD-high-CU**	**CD-low-CU**	**Control**	**Statistic**
Affective perspective-taking. *Mdn *(*IQR*)	4.5 (3)_a_	3 (2)_b_	5(1.50)_c_	*χ*^2^_(2,122) _= 28.25 **
Cognitive perspective-taking. *Mdn *(*IQR*)	5 (2.25)_a_	4 (2)_b_	5(2)_a_	*χ*^2^_(2,122) _= 15.92 **

*Note: Mdn*: Median; *IQR *= Interquartile Range; Medians in the same row that do not share subscripts differ at *p *< .05 in the Mann-Whitney *U *procedure.

**p < .001

A different pattern was observed in the analysis of cognitive perspective-taking across the three groups. As presented in Table 2 the three groups differed in cognitive perspective-taking. Pairwise comparisons showed that the *CD-low-CU *group was outperformed by both control (*z *= -3.40, *p *< .001) and *CD-high-CU *(*z *= -2.54, *p *< .01) groups. *CD-high-CU *and control groups did not differ significantly in cognitive perspective-taking.

In a follow up stage the data of the limited data on girls were excluded and an analysis was performed solitarily on the boys' data. This analysis revealed patterns that were analogous to the results before exclusion of the data on girls. On affective perspective-taking the *CD-low-CU *boys were outperformed by both the control (*z *= -5.26, *p *< .001) and the *CD-high-CU *(*z *= -2.01, *p *< .03) boys. *CD-high-CU *boys were outperformed by controls (*z *= -2.41, *p *< .02). On cognitive perspective-taking the *CD-low-CU *boys were outperformed by both, the control (*z *= -3.69, *p *< .001) and the *CD-high-CU *(*z *= -2.37, *p *< .02) boys. *CD-high-CU *and control boys did not differ significantly in cognitive perspective-taking.

## Discussion

Present findings indicated that *CD-low-CU *children were inferior in cognitive perspective-taking relative to controls and to *CD-high-CU *children who display a more severe pattern of antisocial conduct. On affective perspective-taking, both CD groups were inferior to controls, and *CD-low-CU *children were inferior to *CD-high-CU*-children. Consequently, the conceptual deficits in affective and/or cognitive perspective-taking that have long been implicated in CD [6,7] found only partial support from present findings. This partial support may help to explain previous contradictory findings that, on the one hand, found an association between persistent antisocial conduct and deficits in perspective-taking [9-12,55,56] and on the other hand, challenged the link between persistent antisocial conduct and perspective-taking deficits [13,24,25]. The present findings suggest that earlier contradictory findings might be linked to a significant variation among CD children, namely, that CD subgroups present differentiated cognitive and affective perspective-taking abilities.

Present data revealed deficits in affective perspective-taking in both CD samples compared with 'typically-developing' comparisons with a significantly greater deficit in the *CD-low-CU *group. These findings extend downwards in age the deficits in affective perspective-taking identified by Cohen and Strayer [55] in clinically identified CD adolescents (aged 14 – 17) and by Waterman [13] in institutionalised CD boys (aged 10 – 12), and upwards the deficits identified by Minde [56] in clinically referred CD preschoolers (aged 4 – 4.5).

Three tentative conclusions may be drawn from the findings of the current study. Firstly, as the *CD-low-CU *children demonstrated deficiencies in both cognitive and affective perspective taking, one possible interpretation is that their inferior affective perspective-taking, relative to both other groups, may derive from their relatively weaker cognitive perspective-taking abilities. If understanding others' emotions depends first on understanding their thoughts, it is arguable that weak cognitive perspective-taking might preclude the possibility of effective affective perspective-taking.

Secondly, the *CD-high-CU *children demonstrated cognitive perspective-taking competence accompanied by deficits in affective perspective-taking. One explanation might be that this group demonstrates an affective-specific deficit, perhaps underlined by (or related to) deficits in emotion processing [36,37] and/or deficits in affective empathy (i.e. capacity for vicarious affective responding [40]. Based on the theoretical assumption that the two dimensions of empathy interact [57], if present preliminary data are replicated, it seems that this group potentially presents a disjunction between purely cognitive (i.e. cognitive perspective-taking) and affective (i.e. vicariously-aroused affect) dimensions of empathy that warrants replication and further exploration for conclusions to be drawn with greater confidence.

Thirdly, given that *CD-high-CU *children have shown inferiority, relative to controls, in affective but not in cognitive perspective-taking, and superiority over *CD-low*-*CU *children in both cognitive and affective perspective-taking, it seems that their superiority over the *CD-low-CU *children derives from a relatively greater capacity in understanding others' thoughts, rather than others' emotions. Given also that *CD-high-CU *children exhibited relatively more severe antisocial behaviour than their *CD-low-CU *counterparts, this interpretation seems difficult to reconcile with the findings of Waterman et al. [13], who found that affective but not cognitive perspective-taking superiorities are related to more serious patterns of antisocial behaviour in CD children. There are, however, two substantial differences between the present study and that of Waterman et al., namely, sample selection and assessment measures. In the present study, a differential design was generated. Two groups of children that met CD criteria were recruited. These groups represented the upper quartile vs. the 50^th ^percentile or lower in terms of the presence or absence of CU traits. Whereas, in the Waterman et al. study, all children attending a class for children with EBD were tested, and the results reported were correlational. Secondly, Waterman et al. assessed cognitive and affective perspective-taking abilities using two distinct tasks. Cognitive perspective-taking was assessed by the Flavell et al.[8] perspective-taking logic task, which mostly taps problem-solving skills rather than cognitive perspective-taking; and affective perspective-taking was assessed with the use of videotaped scripts portraying social interactions in which children had to identify the portrayed emotion. In contrast, in the present investigation, cognitive and affective perspective-taking were assessed within the same context, around the same social situation. The advantage of the same context task is that cognitive and affective perspective-taking are interdependent and it therefore allows the detection of a possible disjunction between the two.

Two more general conclusions also emerge. First, as *CD-high-CU *children did not show deficits in cognitive perspective-taking relative to controls, cognitive perspective-taking competency does not prevent antisocial behavior. Similar conclusions have been reached by other empirical investigations utilising normative samples [25,27]. Some investigators [19] have gone further to argue that, in certain children with antisocial behaviour (i.e. bullies), perspective-taking superiorities are associated with greater antisocial acts. Present data have shown that *CD-high-CU *children exhibit relatively more severe antisocial behaviour than their *CD-low-CU *counterparts. Similarly, in a normative sample, Silvern and colleagues [28] reported that, among 10–11 year-old boys, superior cognitive perspective-taking was associated with relatively more severe antisocial behaviour. However, present data are only cross-sectional, so aetiology cannot be established. It is possible that superior cognitive perspective-taking and relatively more severe antisocial conduct in certain CD children develop contemporaneously without direct causal links. Similarly, the relative deficit in both affective and cognitive perspective-taking in *CD-low-CU *children should not be interpreted as implying either a causal relationship or that inferior perspective-taking can solely account for the patterns of behaviour they exhibit. Furthermore, perspective-taking deficits are not restricted to CD children. Poorer perspective-taking performance is characteristic of other clinical child and adolescent samples (e.g. Pervasive Developmental Disorder-PDD, Non-verbal Learning Disorder-NLD, Hyperlexia) [58], and this deficit does not lead them to antisocial conduct. Significant variations in perspective-taking are also seen in normative child and adolescent samples.

Previous research on emotion has documented decreased orienting to negative emotional stimuli in *CD-high-CU *children, and increased orienting to negative emotional stimuli in *CD-low-CU *children [36], as well as deficits in vicarious affective responsiveness [40] in *CD-high-CU *but not in *CD-low-CU *children. Taken together with present findings, these results suggest substantial differences in emotion related processing and responding across CD subgroups that warrant further investigation.

Despite its significant findings, the present investigation should be placed in the context of several important limitations. First, although the emerging body of empirical findings support the validity of the CU subscale in assessing these traits in a theoretically meaningful manner, the internal consistency of this subscale is rather low. A second methodological issue concerns the assessment of perspective-taking, and the extent to which present findings will withstand tests of ecological validity. It may be, for instance, *that CD-high-CU *children do not fail to understand others' cognitive perspective in the context of an empirical task, but, in ambiguous real life situations, the interplay of various interactive, dispositional and situational factors might make them fail to do so. Crick and Dodge [59], for instance, have reported that it is in ambiguous situations that CD children attribute hostile intent to others.

There are also some problems inherent in rating scales that apply to the current investigation. Informants' ratings were primarily based upon overtly-observed behaviour, and in some cases both informants were drawn from the single setting of the child's residential intervention unit. The informants judged the degree to which children manifested certain behaviour traits, and their relative judgements would necessarily be based on a comparison of the children within their own institution. Comparatively speaking, children from the residential EBD institution might have had greater conduct problems than children in EBD day schools, resulting in differing bases of comparison. Ideally, multiple informants from different settings should be employed when assessing child psychopathology [60]. Ratings can also be affected by the informant's preconceptions about the child [61] or the informant's mental state [48]. For instance, an informant's general impression of a particular child might encourage a tendency to rate that child high or low on all the scales; or an informant who is temperamentally overanxious might be more sensitive to or judgmental of children's anxieties or behaviours.

With respect to the measurement of verbal ability a verbal intelligence test to control for variations in language ability was deemed more appropriate than a test of general intelligence, or of non-verbal intelligence. Tests of verbal ability are standard practice in similar studies of sociocognition. However it is conceivable that other aspects of cognitive functioning (e.g. memory, causal reasoning, social information processing, etc) may affect measures of children's sociocognitive awareness. A battery of such tests was beyond the scope of the present study, but should be considered in future research.

On the question of the generalisability of the results, both CD groups consisted predominantly of boys, so the findings should not be considered as generalisable to CD girls. Whilst gender differences are generally not noted in social cognition [22], data are not unanimous [28].

## Conclusion

In conclusion, present findings indicate that deficits in cognitive perspective-taking that have long been implicated in CD appear to be characteristic of a subset of CD children, namely, *CD-low-CU *children. In contrast, affective perspective-taking deficits characterise both CD subgroups, but these defects seem to be following diverse developmental paths that warrant further investigation. In *CD-low-CU *children, affective perspective-taking deficits are underlined by cognitive perspective-taking deficits while in *CD-high-CU *children affective perspective-taking deficits are unaccompanied by cognitive perspective-taking deficits, a finding which is suggestive of dissociation of affective and cognitive perspective-taking in *CD-high-CU *children. These findings have theoretical implications in the taxonomy of aggression and antisocial conduct, since they suggest that a subtyping of CD with reference to CU traits should be considered. Further, present findings have important clinical implications since they provide support to the conjecture that CD children comprise an heterogeneous group with diverse deficits. Such findings suggest that treatment approach needs be individualised to the specific deficits of the each CD child, rather than applying the 'most successful intervention' across all CD children uniformly.

## Abbreviations

CD: Conduct Disorder; CU: Callous-Unemotional; CD-high-CU: CD children elevated on CU traits; CD-low-CU: CD children low on CU traits; EBD: Emotional and Behaviour Difficulties; SES: Socioeconomic Status; *M:* Mean; *Mdn:* Median; IQR: Interquartile Range; SD: Standard Deviation; ADHD: Attention Deficit and Hyperactive Disorder; ODD: Oppositional Defiant Disorder; PTSD: Post Traumatic Stress Disorder

## Competing interests

The authors declare that they have no competing interests.

## Authors' contributions

The greater bulk of this research was carried out by the first author in partial fulfillment of the degree of PhD at the University of Strathclyde, UK. XAH conceived and designed the study, collected, analysed and interpreted the data, drafted and revised the manuscript. DW supervised all the phases of the research, approved the design and assisted with the revision of the drafts. Both authors read and approved the final manuscript.
